# Molecular characterization and genetic structure of the Nero Siciliano pig breed

**DOI:** 10.1590/S1415-47572010005000075

**Published:** 2010-12-01

**Authors:** Anna Maria Guastella, Andrea Criscione, Donata Marletta, Antonio Zuccaro, Luigi Chies, Salvatore Bordonaro

**Affiliations:** 1 Dipartimento di Scienze Agronomiche, Agrochimiche e delle Produzioni Animali, Università degli Studi di Catania, CataniaItaly; 2 Dipartimento di Scienze e Tecnologie Agro-Forestali ed Ambientali, Università degli Studi Mediterranea di Reggio Calabria, Reggio CalabriaItaly

**Keywords:** cluster analysis, coancestry, intra-breed genetic variation, microsatellites, Nero Siciliano pig breed

## Abstract

Nero Siciliano is an autochthonous pig breed that is reared mainly in semi-extensive systems in northeastern Sicily. Despite its economic importance and well-appreciated meat products, this breed is currently endangered. Consequently, an analysis of intra-breed variability is a fundamental step in preserving this genetic resource and its breeding system. In this work, we used 25 microsatellite markers to examine the genetic composition of 147 unrelated Nero Siciliano pigs. The total number of alleles detected (249, 9.96 per *locus*) and the expected heterozygosity (0.708) indicated that this breed had a high level of genetic variability. Bayesian cluster analysis showed that the most likely number of groups into which the sample could be partitioned was nine. Based on the proportion of each individuals genome derived from ancestry, pigs with at least 70% of their genome belonging to one cluster were assigned to that cluster. The cluster size ranged from 7 to 17 (n = 108). Genetic variability in this sub-population was slightly lower than in the whole sample, genetic differentiation among clusters was moderate (*F*_*ST*_ 0.125) and the *F*_*IS*_ value was 0.011. NeighborNet and correspondence analysis revealed two clusters as the most divergent. Molecular coancestry analysis confirmed the good within-breed variability and highlighted the clusters that retained the highest genetic diversity.

## Introduction

In recent decades, many livestock breeds have experience a severe loss of biodiversity that has markedly affected animal production systems, especially in marginal areas. Attempts to reverse this negative trend have led to research in the preservation and exploitation of local animal breeds, with efforts to identify and reintroduce potentially important genetic traits that have been overlooked by globalized production systems. Autochthonous breeds require careful molecular and morphological characterization that takes into account potential influences of the environment in which they were originally developed and adapted.

Nero Siciliano is an ancient black pig breed that originated in Sicily and has always been reared in extensive and semi-extensive systems on this island. Currently, most of the farms that rear autochthonous black pigs (1800 pigs, 70% of the entire population for this breed) are located mainly in the mountainous area of Nebrodi in northeastern Sicily. Black pigs are rustic animals that thrive on roughage and a limited food supply, in addition to being resistant to diseases in harsh conditions. This breed still retains its distinctiveness thanks to the geographical and orographical characteristics of the island and breeding area, but runs a high risk of losing its original traits because of the lack of a suitable plan to safeguard and exploit its production.

Nero Siciliano pigs grow slowly and yield tasty meat and fat (Pugliese *et al.*, 2003) used to produce high quality meat, including salami and cured ham that are very appreciated by local consumers. The creation of a Protected Designation of Origin (PDO) label for Nero Siciliano meat and other related products has helped to safeguard and preserve this breed, and has led to an important increase (21%) in the number of farms and in sow rearing (24%) in the last three years. Despite this renewed interest of breeders and consumers, only 850 breeding sows are currently being reared and the Nero Siciliano breed is included in the list of endangered autochthonous breeds.

Genetic characterization is a fundamental prerequisite for managing genetic resources. A recent morphometric analysis of Nero Siciliano pigs involving 13 body measurements highlighted the low-medium size of this breed when compared with other Italian breeds (Guastella *et al.*, 2009). Genetic variability has also been assessed based on the use of different genetic markers (Russo *et al.*, 2004; D'Alessandro *et al.*, 2007; Davoli *et al.*, 2008). In particular, microsatellite markers have been particularly useful for quantifying genetic variation within and among several European pig breeds, including Nero Siciliano (SanCristobal *et al.*, 2006).

The aim of this study was to use microsatellite markers to assess the genetic variability and genetic structure of Nero Siciliano pigs reared in the Nebrodi mountains, in order to provide suitable data for conservation strategies.

## Material and Methods

###  Sample collection and microsatellite analysis

A representative sample of 147 Nero Siciliano pigs (22 boars and 125 sows) was selected from 22 farms in 11 communes (Alcara Li Fusi, Brolo, Capizzi, Caronia, Floresta, Longi, Mirto, Sanfratello, San Salvatore di Fitalia, Raccuja and Tortorici) in the Nebrodi area; first- and second-degree relatives were avoided. The Nebrodi area is part of a natural Park (37°50'-38°9' N; 14°26'-14°54' E) located 100-1700 m above sea level, and is where the Nero Siciliano breed originated and is still extensively bred. The sample size ranged from 2 to 15 pigs per farm, depending on the herd size. Only pigs that met the morphological standard for the breed were sampled, and digital photographs of each animal were taken. Farmers were also asked about the breeding strategies that they employed to select animals with an exclusively native Sicilian germplasm: farms in which there had been recent crossbreeding with commercial breeds were excluded from the sampling.

For each pig, 10 mL of peripheral blood was collected in K3-EDTA tubes. DNA was extracted from blood using the commercial Illustra blood genomic Prep Mini Spin kits (GE Healthcare, Little Chalfont, UK). Genetic characterization was done with a set of 25 microsatellite markers (Table S1) chosen from a list maintained by the Pig Biodiversity project and USDA MARC database. The markers were chosen based on preliminary data about their degree of heterozygosity and polymorphism obtained from small samples of Nero Siciliano and other local Italian pig populations. Six PCR multiplex reactions and two single PCR reactions were done to amplify the microsatellites according to standard protocols. The amplicons (2 μL) were mixed with 4 μL of loading buffer containing formamide and 350 TAMRA as an internal size standard (Applied Biosystems, Warrington, UK). Individual genotypes were determined with an ABI PRISM^®^ 377 sequencer equipped with Genescan Analysis^®^ v.3.1.2 and Genotyper^®^ v. 2.5 softwares (Applied Biosystems, Foster City, CA, USA).

###  Statistical analysis

Individual multilocus genotypes were analyzed by Molkin v. 3.0 software (Gutierrez *et al.*, 2005) to calculate the main parameters of genetic variability. For each *locus* and group of pigs, the allele frequencies, private alleles (A_p_), effective number of alleles (A_e_), and observed (H_o_) and expected (H_e_) heterozygosities were calculated. Molecular coancestry coefficients and kinship distances (*D*_*k*_) weighted by polymorphic information content (PIC) were also assessed. In addition, the contribution of the different groups of pigs to the overall or total diversity was also inferred, according to Caballero and Toro (2002) and Petit *et al.* (1998).

Genepop v. 4.0 (Rousset, 2007) was used to perform the score test for Hardy-Weinberg equilibrium (Rousset and Raymond, 1995) per *locus* using a Markov chain algorithm implemented with 10,000 dememorizations, 200 batches and 5000 iterations per batch. The presence of null alleles was tested with MICRO-CHECKER v. 2.2.3 (Van Oosterhout *et al.*, 2004), using Bonferroni adjustments.

FSTAT v.2.9.3 software (Goudet, 2001) was used to estimate the *F*_IT_, *F*_ST_ and *F*_IS_ statistics (Weir and Cockerham, 1984) and their significance was inferred by methods based on randomisation. Multiple tests of significance were corrected by the sequential Bonferroni method (Rice, 1989).

The model-based approach proposed by Falush *et al.* (2003) in the software STRUCTURE v.2.2 was used to assess the genomic clustering of the sample. Individual pigs were probabilistically assigned to two or more subpopulations on the basis of their multilocus genotype, assuming that they were admixed. As suggested by the authors, the admixture model associated with the option of correlated allele frequencies was used to infer the population structure. The run length was set to 100,000 burn-ins followed by 100,000 iterations. This setting produced consistent estimations that were not significantly altered by a longer burn-in or Markov chain Monte Carlo (MCMC). The range of possible clusters (*K*) tested was from 1 to 15, and 10 runs were done for each *K*. CLUMPP software (Jakobsson and Rosenberg, 2007) was subsequently used to find the optimal alignment of the 10 replicate cluster analyses of the same *K*. The similarity coefficient G', which is also a measure of the constancy over runs, was used to define the population structure. The mean membership matrix across replicates was plotted with the program DISTRUCT (Rosenberg, 2004). The method reported by Evanno *et al.* (2005) was also used to estimate the most likely number of *K* that explained the sample structure.

Reynolds' pairwise distances (Reynolds *et al.*, 1983), used to assess the genetic relationship between clusters, were calculated with the software Phylip v. 3.67 (Felsenstein, 2005). A multivariate method of correspondence analysis allowed the simultaneous representation of inferred clusters. GENETIX v.4.05 software (Belkhir *et al.*, 2004) was used to spatially plot clusters and individuals based on the allele frequencies of all *loci* and a correspondence analysis in which the Chi-square distances served to judge the proximity of the clusters.

## Results

Nero Siciliano pigs showed high genetic variability (Table 1). Two hundred and forty-nine alleles were detected, with 5 (*locusS0026*) to 19 (*locusS0005*) alleles per *locus* and an average number per *locus* (9.96) that was fairly high. The effective number of alleles (A_e_), which takes into account the expected heterozygosity (H_e_), showed that *S0005* was the most polymorphic *locus* and *SW951* the least polymorphic. The expected heterozygosity was higher than that observed at each *locus* and ranged from 0.245 to 0.907 (mean: 0.708). The estimated polymorphic information content (PIC) ranged from 0.236 at *SW951* to 0.901 at *S0005* (data not shown). Overall, Nero Siciliano pigs showed Hardy-Weinberg disequilibrium, with significant deviations from equilibrium being observed for 10 microsatellites (Table 1). The presence of null alleles, inferred for five *loci* (*S005*, *SW911*, *SW1873*, *SW1556*, *SW2038*), could explain the excess of homozygotes and the deviation from genetic equilibrium at these *loci*. The heterozygote deficiency within populations (*F*_IS_) was significant for nine *loci*. The overall *F*_IS_ coefficient for the *loci* was 0.109, indicating a significant (p < 0.001) excess of homozygotes in the whole sample.

The level of population structuring was quite high. Model-based clustering of the microsatellite genotypes revealed that the likelihood variance of the observed data initially increased by the predefined number of clusters to reach a peak value at *K* = 9 and then decreased. As indicated by Pritchard *et al.* (2000), nine is the smallest value of *K* that captures the major structure in the dataset. Based on the method proposed by Evanno *et al.* (2005), two clear peaks at *K* = 9 and *K* = 2 (the latter being particularly indicative of a very low likelihood variance) were observed in the Δ*K* distribution. The highest values of the similarity coefficient G' (> 95%) were detected at *K* = 2, *K* = 7 and *K* = 9. Based on these three approaches, we assumed *K* = 9 to be the most likely number of clusters.

Based on the average matrix of membership (data not shown), animals with at least 70% of their genome belonging to one cluster were assigned to that cluster. The cluster size ranged from 7 to 17 (for a subsample of 108 of the original 147 pigs). Table 2 shows the genetic parameters of diversity for the nine clusters and the subsample of 108 pigs. The average genetic differentiation among inferred clusters was moderate (*F*_ST_ 0.125; p = 0.001). The genetic variability of this subsample was still high with the overall *F*_IS_ value (0.011) not significantly different from zero, indicating frequent random mating. The observed and expected heterozygosities were slightly lower than in the whole sample of 147 pigs.

Among the 22 farms sampled, nine were highly homogenous, with > 70% of the genome belonging to a cluster. In contrast, animals from three farms that were particularly active in fattening pigs for meat production and pork products showed a wide genome distribution among clusters. Some clusters (*K7*, *K5* and *K3*) consisted of individuals from single farms, whereas two (*K9* and *K4*) consisted of pigs from different herds reared in the same area.

The rarefacted number of alleles (Ar), which measures the contribution of alleles weighted by the sample size, ranged from 3.01 (*K*7) to 4.40 (*K*1). Thirty-three private alleles were detected in the model-inferred group of pigs. Each cluster was characterized by at least one unique allele: high frequencies were observed for alleles *SW1695* (167 bp), *SW1928* (85 bp) and *S0005* (258 bp) in *K*3 (0.39), *K*7 (0.36) and *K*2 (0.25), respectively.

NeighborNet built on Reynolds' distances revealed a close linkage among most of the clusters: *K*7 and *K*2 were the most divergent in terms of average distance, with values of 0.190 and 0.182, respectively. The lowest pairwise distance was between *K*6 and *K*8, and the highest between *K*7 and *K*5 (data not shown).

**Figure 1:**
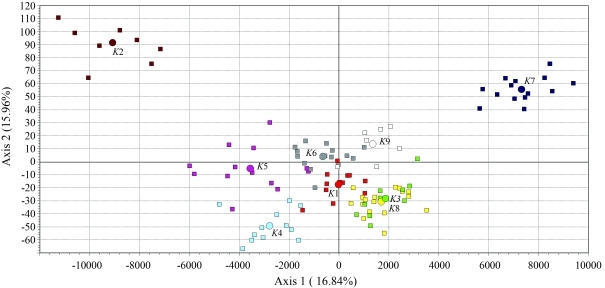
Summary of individual and cluster positions according to the first and second axis of dispersion in correspondence analysis. Circles and squares indicate clusters and individuals, respectively.

Correspondence analysis provided an alternative spatial representation of individuals and clusters scattered in the metric space (Figure 1). The first two axes contributed almost equally to the total inertia (16.84% and 15.96%, respectively). With regard to the dispersion in the first and second axes, *S0017* (155 bp), *SW1928* (85 bp), *SW240* (122 bp) and *S0017* (155 bp) were the most important alleles, each with a contribution > 5.7%.

The spatial dispersion of clusters and related individuals was determined mainly by four alleles that had very different frequencies among clusters, and by high frequency alleles detected exclusively in a given cluster. In particular, allele *SW1928* (85 bp), which was found only in *K*7 at a high frequency (35.7%), contributed significantly to dispersion in the first and second axes (3.8% and 2.4%, respectively); an additional three alleles contributed more than 5% to dispersion in the first two axes and occurred at very high frequencies, *i.e.*, 50% for *SW240* (122 bp) in *K*2, 56% for *S0017* (155 bp) in *K*2 and 82% for *SW1873* (136 bp) in *K7*.

Molecular coancestry in each cluster ranged from 0.287 in *K*1 to 0.389 in *K*7. Self-coancestry and the inbreeding coefficient showed the same trend: the lowest values were detected in *K6*, the highest in *K3*. Kinship distances ranged from 0.277 to 0.361. The contribution to the diversity of the whole sample is shown in Table 2. Cluster 2 contributed the most to the total genetic diversity (CGD) and *K*8 the least, when assessed according to Caballero and Toro (2002). However, when the rarefacted number of alleles was considered, clusters *K1* and *K7* provided the highest and lowest contributions (CAr), respectively (Petit *et al.*, 1998).

## Discussion

Nero Siciliano pigs showed considerable genetic variability and diversity, expressed in terms of the number of alleles per *locus* (NA) and expected heterozygosity (H_e_): the values for these parameters were higher than for several European breeds (NA = 4.5 on average, H_e_ = 0.43-0.68), including Iberian breeds (NA = 3.44-5.86, H_e_ = 0.46-0.64) (Martinez *et al.*, 2000), seven native and commercial breeds reared in Portugal (NA = 3.77-6.27, H_e_ = 0.49-0.69) (Vicente *et al.*, 2008), and a population of Nero Siciliano pigs (NA = 6.70, H_e_ = 0.67) studied by SanCristobal *et al.* (2006). These values were also greater than those reported for Korean native pigs (NA = 3.44, H_e_ = 0.49) (Kim *et al.*, 2005), Cuban Criollo pigs (NA = 8.2, H_e_ = 0.65) (Martinez *et al.*, 2005) and most Chinese breeds (NA = 6.25-11.55, H_e_ = 0.57-0.77) (Li *et al.*, 2004)*.*

The number of effective alleles (4.53) was higher than in European breeds (2.74 on average) and Nero Siciliano pigs (4.03) studied by SanCristobal *et al.* (2006) and higher than or comparable to those reported for several Chinese breeds (1.69-5.62) (Li *et al.*, 2004; Fang *et al.*, 2005), except for Lingao (4.76; Fang *et al.*, 2005) and Nang Yang Black, Sheng Xian Spotted and Hai Nan (5.12, 5.21 and 5.62, respectively) (Li *et al.*, 2004). Conversely, the number of effective alleles and expected heterozygosity in Nero Siciliano pigs was lower than in the Indian Ankamali breed (Ae = 5.34, H_e_ = 0.83) (Behl *et al.*, 2006).

The significant deviation from Hardy-Weinberg equilibrium observed at 10 *loci* may reflect the presence of null alleles at some *loci* and the Wahlund effect, *i.e.*, a reduction in the observed heterozygosity that occurs in subdivided populations. Genetic substructures in Nero Siciliano pigs apparently resulted from different management strategies across herds and inbreeding within herds. In the Nebrodi area, farmers use their own boars for a year or more, with only a limited exchange of animals among farms and herds. The within-group inbreeding values (*F*_IS_) support this hypothesis (Table 2).

The unsupervised analysis of population structure detected nine homogenous groups in the sample analyzed and determined the corresponding fraction of the individual's genome derived from ancestry in each cluster. Cluster *K*1 showed the highest variability in the number of alleles and heterozygosity, whereas *K*7 showed the least. These results agreed with the values for molecular coancestry and were confirmed by analysis of the contribution to the total genetic diversity estimated from the allelic richness.

The negative *F*_IS_ values detected in clusters *K*2 and *K*5, which represent two closed-cycle farms, probably reflected the proper selection of breed animals among the farms sampled. In these farms, which sell piglets of different ages for production or reproduction, the systematic exchange of genetic resources that occurs between breeding and processing herds probably helps to maintain a high level of diversity. Molecular data confirmed that these two farms had good mating management programs that ensured high genetic variability.

Correspondence analysis provided a more interesting and informative spatial representation of the relationships among the nine clusters with respect to the network built on genetic distances. Clusters *K*2 and *K*7 represented the two most distinct group of pigs and accounted for 14.2% and 12.7% of the inertia, respectively. Cluster 7 represented a single herd. The farm in question is interesting because it is the only one in the Nebrodi area in which the recessive mutation in the RYR1 (*Ryanodine Receptor 1*) *locus* responsible for the Porcine Stress Syndrome (PSS) has been reported (Matassino *et al.*, 2007); ten of the 14 pigs in this cluster were carriers of this recessive allele. Despite their morphological traits and recent successful breeding history, these pigs retain the traces of a possible accidental introgression of other commercial breeds. In addition, the low values for effective alleles and expected heterozygosity in *K*7 may reflect mating between closely related or inbred animals. Cluster 2 also represented a single farm that was among the first to rationally manage the short chain cycle of breeding-processing-resale. The spatial distribution of *K*2 pigs and the high contribution of this cluster to overall diversity support the hypothesis that the present herd was derived from different genetic lineages that were incorporated to control the level of inbreeding.

Mitochondrial DNA haplotype analysis has shown that modern European pig breeds belong to a general European cluster (Larson *et al.*, 2005). The populations are often heavily structured and more than half of the European pig diversity can be assigned to local breeds (Ollivier *et al.*, 2005). The results described here indicate a high degree of genetic variability in Nero Siciliano pigs, as previously reported (SanCristobal *et al.*, 2006; Davoli *et al.*, 2007), despite the limited population size of this breed. This is a rather frequent condition for populations reared in extensive systems where there is no systematic selection and planned mating. Part of the observed heterogeneity may have originated from accidental crosses with wild boars (Ollivier *et al.*, 2005) and limited introgression with other Italian breeds (Neapolitan and Casertana), as well as with Iberian breeds and commercial pigs (Russo *et al.*, 2004).

As shown here, the genetic profile established by using neutral markers and the analysis of intra-population structure provided a general outline of the breeding systems applied to Nero Siciliano pigs in the Nebrodi area. The model-based approach identified homogenous groups in the sample, some of which coincided with specific farms and others with breeding areas. Our results indicate that molecular data can be helpful in the selection of parental stocks and planned matings as part of strategies to preserve and restore the rational breeding of black pigs. Boars belonging to cluster *K7* need to be screened for the PSS syndrome gene and possibly excluded from selection programs in order to eliminate the PSS mutated gene and related PSE (pale, soft, exudative) defect from the population gene pool. Individuals belonging to different clusters could be used in planned matings to maintain a good level of genetic variability and rusticity (stress-resistance) and avoid excessive inbreeding. On the other hand, pigs sharing the same clusters and chosen based on their individual multilocus genotypes may be used in planned matings to preserve the most typical traits in this autochthonous population. Nero Siciliano pigs belonging to the most divergent cluster (*K2*) and pigs not included in the most homogenous subpopulation of 108 animals need to be incorporated into selection schemes in order to counterbalance any increase in inbreeding. Such approaches should help to preserve the Nero Siciliano breed and ensure the production of high quality products for local and national markets.

## Supplementary Material

The following material is available online for this article:

Table S1 Multiple (M) and single (S) PCR, locus name and chromosomal location, and primer sequences and dyes used in PCR

This material is available as part of the online article from http://www.scielo.br/gmb.

## Figures and Tables

**Table 1:** Number of observed (NA) and effective (A_e_) alleles, observed (H_o_) and expected heterozygosity (H_e_), and F_IS_ per *locus*.

Locus	NA	A_e_	H_o_	H_e_	F_IS_
*SW240*	16	5.66	0.738	0.823	0.107*
*SW857*	9	5.24	0.762	0.809***	0.061
*S0155*	7	3.25	0.632	0.692	0.091
*S0101*	8	3.82	0.732	0.738	0.012
*S0005*	19	10.78	0.782	0.907**	0.141***
*S0355*	8	1.94	0.429	0.484	0.117
*SW936*	11	3.85	0.676	0.740	0.091
*SW72*	9	4.02	0.731	0.751	0.030
*SW911*	7	3.97	0.600	0.748***	0.201**
*S0228*	7	1.68	0.380	0.403	0.060
*SW1928*	8	3.65	0.695	0.726	0.046
*SW1873*	13	6.73	0.667	0.851**	0.221***
*SW1695*	14	8.75	0.810	0.886*	0.089
*SW1556*	6	2.08	0.293	0.519***	0.439***
*SW1370*	14	6.14	0.738	0.837*	0.122*
*SW1035*	12	2.37	0.500	0.578*	0.138*
*SWR153*	8	3.20	0.600	0.688	0.131
*SW2038*	11	7.14	0.760	0.860	0.119**
*S0017*	10	2.62	0.558	0.618	0.101
*SW1823*	12	7.53	0.807	0.867	0.073
*S0026*	5	2.29	0.521	0.562	0.077
*SW24*	11	5.45	0.782	0.817	0.045
*SW951*	6	1.32	0.221	0.245*	0.102
*SW632*	12	6.27	0.828	0.840	0.019
*S0090*	6	3.51	0.603	0.715*	0.160**
Average	9.96	4.53	0.634	0.708***	0.109***

Deviations from H-W equilibrium and significance of the F_IS_ values are indicated by asterisks (*p < 0.05, **p < 0.01 and ***p < 0.001).

**Table 2:** Number of heads, average number of observed (NA) alleles per *locus*, private alleles (A_p_) with the highest allele frequency in parentheses, observed (H_o_) and expected (H_e_) heterozygosities, and F_IS_ values per cluster and for the subsample of 108 pigs. The contribution of allelic richness (CAr) and gene diversity (CGD) per cluster to the diversity of the subsample is also shown.

Cluster	Heads	NA	A_p_	H_o_	H_e_	F_IS_	CAr	CGD
K1	13	6.32	4 (11.5)	0.675	0.669	0.033	2.059	-1.044
K2	8	3.88	5 (25.0)	0.670	0.600	-0.046	1.154	-1.136
K3	10	4.48	5 (38.9)	0.574	0.621**	0.137*	1.048	-0.498
K4	12	4.88	2 (16.7)	0.568	0.601*	0.106*	0.798	-0.082
K5	12	4.16	1 (8.3)	0.627	0.556	-0.084	-0.724	0.319
K6	15	5.72	4 (13.3)	0.683	0.642	-0.029	0.814	-0.354
K7	14	3.72	4 (35.7)	0.543	0.511	-0.026	-1.535	0.332
K8	17	5.76	4 (11.8)	0.629	0.616	0.002	-0.365	0.747
K9	7	4.12	4 (14.3)	0.632	0.607	0.032	0.456	-0.278
Total	108	9.52	33	0.622	0.706	0.011	-	-

## References

[Behletal2006] Behl R., Sheoran N., Behl J., Vijh R.K. (2006). Genetic analysis of Ankamali pigs of India using microsatellite markers and their comparison with other domesticated Indian pig types. J Anim Breed Genet.

[Belkhiretal2004] Belkhir K., Borsa P., Chikhi L., Raufaste N., Bonhomme F. (2004). GENETIX 4.05, Logiciel sous Windows TM pour la Génétique des Populations.

[CaballeroandToro2002] Caballero A., Toro M.A. (2002). Analysis of genetic diversity for the management of conserved subdivided populations. Conserv Genet.

[DAlessandroetal2007] D'Alessandro E., Fontanesi L., Liotta L., Davoli R., Chiofalo V., Russo V. (2007). Analysis of the MC1R gene in the Nero Siciliano pig breed and usefulness of this locus for breed traceability. Vet Res Commun.

[Davolietal2007] Davoli R., Zambonelli P., San-Cristobal M., Scotti E., Fontanesi L., Colombo M., Dall'Olio S., Braglia S., Russo V., Nanni Costa L., Zambonelli P., Russo V. (2007). SNPs and microsatellite markers for genetic diversity study in Italian pig breeds. Proceedings of the 6^th^ International Symposium on the Mediterranean Pig.

[Evannoetal2005] Evanno G., Regnaut S., Goudet J. (2005). Detecting the number of clusters of individuals using the software STRUCTURE: A simulation study. Mol Ecol.

[Falushetal2003] Falush D., Stephens M., Pritchard J.K. (2003). Inference of population structure: Extensions to linked loci and correlated allele frequencies. Genetics.

[Fangetal2005] Fang M., Hu X., Jiang T., Braunschweig M., Hu L., Du Z., Feng J., Zhang Q., Wu C., Li N. (2005). The phylogeny of Chinese indigenous pig breeds inferred from microsatellite markers. Anim Genet.

[Guastellaetal2009] Guastella A.M., Bordonaro S., Zuccaro A., Marletta D., Criscione A., D'Urso G. (2009). Morphological and genetic characterization of Nero Siciliano pig population reared in the Nebrodi area. Ital J Anim Sci.

[Gutierrezetal2005] Gutiérrez J.P., Royo L.J., Álvarez I., Goyache F. (2005). MolKin v. 2.0: A computer program for genetic analysis of populations using molecular coancestry information. J Hered.

[JakobssonandRosenberg2007] Jakobsson M., Rosenberg N.A. (2007). CLUMPP: A cluster matching and permutation program for dealing with label switching and multimodality in analysis of population structure. Bioinformatics.

[Kimetal2005] Kim T.H., Kim K.S., Choi B.H., Yoon D.H., Jang G.W., Lee K.T., Chung H.Y., Lee H.Y., Park H.Y., Lee J.W. (2005). Genetic structure of pig breeds from Korea and China using microsatellite loci analysis. J Anim Sci.

[Larsonetal2005] Larson G., Dobney K., Albarella U., Fang M., Matisoo-Smith E., Robins J., Lowden S., Finlayson H., Brand T., Willerslev E. (2005). Worldwide phylogeography of wild boar reveals multiple centers of pig domestication. Science.

[Lietal2004] Li S.J., Yang S.H., Zhao S.H., Fan B., Yu M., Wang H.S., Li M.H., Liu B., Xiong T.A., Li K. (2004). Genetic diversity analyses of 10 indigenous Chinese pig populations based on 20 microsatellites. J Anim Sci.

[Martinezetal2000] Martinez A.M., Delgado J.V., Rodero A., Vega-Pla J.L. (2000). Genetic structure of the Iberian pig breed using microsatellites. Anim Genet.

[Martinezetal2005] Martinez A.M., Pèrez-Pineda E., Vega-Pla J.L., Barba C., Velàzquez F.J., Delgado J.V. (2005). Genetic characterisation of the Cuban Creole pig with microsatellites. Arch Zootec.

[Matassinoetal2007] Matassino D., Bordonaro S., Castellano N., Guastella A.M., Incoronato C., Monaco F., Occidente M., Pane F., Barone C.M.A., Nanni Costa L., Zambonelli P., Russo V. (2007). CRC *locus* screening in some Italian pig ancient autochthonous genetic types (AAGTs). Preliminary results. Proceedings of the 6^th^ International Symposium on the Mediterranean Pig.

[Ollivieretal2005] Ollivier L., Alderson L., Gandini G.C., Foulley J.L., Haley C.S., Joosten R., Rattink A.P., Harlizius B., Groenen M.A.M., Amigues Y. (2005). An assessment of European pig diversity using molecular markers: Partitioning of diversity among breeds. Conserv Genet.

[Petitetal1998] Petit R.J., El Mousadik A., Pons O. (1998). Identifying populations for conservation on the basis of genetic markers. Conserv Biol.

[Pritchardetal2000] Pritchard J.K., Stephens M., Donnely P. (2000). Inference of population structure using multilocus genotype data. Genetics.

[Puglieseetal2003] Pugliese C., Madonna G., Chiofalo V., Margotta S., Acciaioli A., Gandini G. (2003). Comparison of the performances of Nero Siciliano pigs reared indoors and outdoors. 1. Growth and carcass composition. Meat Sci.

[Reynoldsetal1983] Reynolds J., Weir B.S., Cockerham C. (1983). Estimation of the coancestry coefficient: Basis for a short-term genetic distance. Genetics.

[Rice1989] Rice W.R. (1989). Analysing tables of statistical tests. Evolution.

[Rosenberg2004] Rosenberg N.A. (2004). Distruct: A program for the graphical display of population structure. Mol Ecol Notes.

[Rousset2007] Rousset F. (2007). Genepop'007: A complete re-implementation of the GENEPOP software for Windows and Linux. Mol Ecol Notes.

[RoussetandRaymond1995] Rousset F., Raymond M. (1995). Testing heterozygote excess and deficiency. Genetics.

[Russoetal2004] Russo V., Fontanesi L., Davoli R., Chiofalo L., Liotta L., Zumbo A. (2004). Analysis of single nucleotide polymorphisms in major and candidate genes for production traits in Nero Siciliano pig breed. Ital J Anim Sci.

[SanCristobaletal2006] SanCristobal M., Chevalet C., Haley C.S., Joosten R., Rattink A.P., Harlizius B., Groenen M.A., Amigues Y., Boscher M.Y., Russell G. (2006). Genetic diversity within and between European pig breeds using microsatellite markers. Anim Genet.

[VanOosterhoutetal2004] Van Oosterhout C., Hutchinson W.F., Wills D.P.M., Shipley P. (2004). MICRO-CHECKER: Software for identifying and correcting genotyping errors in microsatellite data. Mol Ecol Notes.

[Vicenteetal2008] Vicente A.A., Carolino M.I., Sousa M.C.O., Ginja C., Silva F.S., Martinez A.M., Vega-Pla J.L., Carolino N., Gama L.T. (2008). Genetic diversity in native and commercial breeds of pigs in Portugal assessed by microsatellites. J Anim Sci.

[WeirandCockerham1984] Weir B.S., Cockerham C.C. (1984). Estimating F-statistics for the analysis of population structure. Evolution.

