# Supplementary material for: Molecular characterization and genetic structure of the Nero Siciliano pig breed
Source: Genet Mol Biol. 2010 Dec 1;33(4):650–6. doi: 10.1590/S1415-47572010005000075 (PMC3036141; doi:10.1590/S1415-47572010005000075)
Supplement: Table S1 — Multiple (M) and single (S) PCR, locus name and chromosomal location, and primer sequences and dyes used in PCR [file gmb-33-4-650-suppl1.pdf]

Table S1 - Multiple (M) and single (S) PCR, locus name and chromosomal location, and primer sequences and dyes used in PCR.

PCR	Locus	Chromosome	Primer sequences (5' - 3')<br>(Forward - F, Reverse - R)	Dye
	S0005	5	F:TCCTTCCCTCCTGGTAACTA<br>R:GCACTTCCTGATTCTGGGTA	FAM
	S0101	7	F:GAATGCAAAGAGTTCAGTGTAGG<br>R:GTCTCCCTCACACTTACCGCAG	TET
	S0155	1	F:TGTTCTCTGTTTCTCCTCTGTTTG<br>R: AAAGTGGAAAGAGTCAATGGCTAT	FAM
M1	*S0355*	15	F:TCTGGCTCCTACACTCCTTCTTGATG<br>R: TTGGGTGGGTGCTGAAAAATAGGA	HEX
	SW240	2	F: AGAAATTAGTGCCTCAAATTGG<br>R: AAACCATTAAGTCCCTAGCAAA	TET
	SW857	14	F:TGAGAGGTCAGTTACAGAAGACC<br>R:GATCCTCCTCCAAATCCCAT	TET
	SW72	3	F: ATCAGAACAGTGCGCCGT<br>R:TTTGAAAATGGGGTGTTTCC	TET
M2	*SW936*	15	F:TCTGGAGCTAGCATAAGTGCC<br>R:GTGCAAGTACACATGCAGGG	HEX
	SW911	9	F:CTCAGTTCTTTGGGACTGAACC<br>R: CATCTGTGGAAAAAAAAAGCC	HEX
	S0228	6	F:GGCATAGGCTGGCAGCAACA<br>R:AGCCCACCTCATCTTATCTACACT	HEX
	SWR1928	7	F:TAGGGTCAGTGCATCCTTCC<br>R:ACGAGAACTCCGAACCCTG	FAM
M3	*SW1873*	7	F:TATAATCTGGTGAACCATCCCC<br>R:ATCAGATGTGCTAATACCCTGC	HEX
	SW1695	2	F: ATAAGGGAAATCAGGCTGAGC<br>R:TCCCAGGAGCTACCATATGC	TET
	SW1556	14	F: TCCCAGCACCTTGATTTTAG<br>R:AGGTTGCTGGAGATAGTGAAGC	HEX
	SW1370	2	F: AGAGCAGTGGTCTGCTAAGATG<br>R: GAATTGCCTAAATTTACTTGTCC	HEX
M4	*SW1035*	16	F:TATGGGGGCCCTAAAAAGAC<br>R: AACGGCCTTAACCTCCTCAG	HEX
	SWR 153	4	F: CCACGTTCTCCTTTTTGAGG<br>R:ATGAGTTGTGGTGTAGGTCGC	FAM
	SW2038	14	F: GCCGAGAAACCCTTCACC<br>R: TAGCCTGTTCAGTGCCACC	TET
M5	*S0017*	8	F:CTAGGAGAAAATCTGAGGTT<br>R:GTTTGAATGGAGGTGCTGTA	FAM
	SW1823	6	F: CAGGTCATTGCTGTAGTGAAGG<br>R: GAGCCTTGGGCTACGTAGTG	HEX
	SW 951	10	F:TTTCACAACTCTGGCACCAG<br>R:GATCGTGCCCAAATGGAC	HEX
M6	*SW 632*	7	F:TGGGTTGAAAGATTTCCCAA<br>R:GGAGTCAGTACTTTGGCTTGA	TET
	S0026	16	F:AACCTTCCCTTCCCAATCAC<br>R:CACAGACTGCTTTTTACTCC	HEX
S1	*SW 24*	17	F:CTTTGGGTGGAGTGTGTGC<br>R:ATCCAAATGCTGCAAGCG	FAM
S2	*S0090*	12	F:CCAAGACTGCCTTGTAGGTGAATA<br>R:GCTATCAAGTATTGTACCATTAGG	FAM

FAM - blue, HEX - green, TET - yellow.
